# Polypharmacy and Health-Related Quality of Life Among US Adults With Arthritis, Medical Expenditure Panel Survey, 2010–2012

**DOI:** 10.5888/pcd13.160092

**Published:** 2016-09-22

**Authors:** Abdulkarim M. Meraya, Nilanjana Dwibedi, Usha Sambamoorthi

**Affiliations:** Author Affiliations: Nilanjana Dwibedi, Usha Sambamoorthi, Department of Pharmaceutical Systems and Policy, School of Pharmacy, West Virginia University, Morgantown, West Virginia.

## Abstract

**Introduction:**

Our objective was to determine the relationship between polypharmacy (treatment with prescription drugs from 6 or more drug classes concurrently) and health-related quality of life (HRQoL) among US adults with arthritis.

**Methods:**

We conducted a retrospective cohort study that used 2-year longitudinal data from the Medical Expenditure Panel Survey to analyze a cohort of 6,132 adults aged over 21 years with arthritis. Measures of HRQoL were the summary scores from the mental component summary (MCS) and physical component summary (PCS) of the 12-item short-form health survey. Unadjusted and adjusted regression models were used to evaluate the association between polypharmacy and HRQoL measures. We used SAS, version 9.4, (SAS Institute Inc) to conduct all analyses.

**Results:**

In unadjusted analyses, adults with arthritis taking prescription drugs from 6 or more drug classes concurrently had significantly lower MCS and PCS scores (β, −3.11, *P* < .001 and β, −10.26, *P* < .001, respectively) than adults taking prescription drugs from fewer than 6. After controlling for the demographic characteristics, number of mental and physical chronic conditions, and baseline MCS and PCS scores, adults taking prescription drugs from 6 or more drug classes concurrently had significantly lower PCS scores (β, −1.68, *P* < .001), than those taking prescription drugs from fewer than 6. However, no significant difference in MCS scores was found between adults taking prescription drugs from 6 or more drug classes concurrently and those taking prescription drugs from fewer than 6 (β, −0.27, *P* = .46).

**Conclusion:**

Polypharmacy is significantly associated with lower PCS scores among adults with arthritis. Because polypharmacy can lead to drug–drug and drug–disease interactions, health care providers need to consider the risk and adopt a cautious approach in prescribing multiple drugs to manage chronic conditions and in choosing therapies to improve HRQoL among adults with arthritis.

## Introduction

Arthritis is one of the most prevalent diseases among adults in the United States (1–3) and is the leading cause of adult disability (3,4). Nearly 52.5 million adults reported having some form of arthritis from 2010 through 2012 (2). Arthritis has a negative impact on health-related quality of life (HRQoL) (5–8). Adults with arthritis have worse HRQoL than those without arthritis (7,8). Arthritis is a greater detriment to HRQoL than other common chronic conditions, such as lung disease, congestive heart failure, diabetes, hypertension, and ischemic heart disease (9). Results from previous clinical trials indicated that pharmacologic treatment of arthritis improved HRQoL among adults with arthritis (5,10,11).

Arthritis-related pharmacotherapy can include multiple drug regimens, including analgesics, anti-inflammatory drugs, corticosteroids, disease-modifying antirheumatic drugs, or biological therapies (12,13). Adults with arthritis may have multiple co-existing conditions (2,14) that could be treated with medication. For example, if a woman with osteoarthritis has 4 other chronic conditions (eg, diabetes, hypertension, chronic obstructive pulmonary disease, osteoporosis), she could be prescribed 12 separate medications based on clinical guidelines (15). Some adults with arthritis may be on multiple arthritis-related medications, and if co-existing conditions are also treated with drugs, these adults may be treated with polypharmacy (treatment with prescription drugs from 6 or more drug classes concurrently).

High rates of polypharmacy have been documented among adults with arthritis (12,13,16). In addition, polypharmacy is often associated with low HRQoL (17–19). Although the association between arthritis and polypharmacy was documented (12,13,16), no study to date has examined the effect of polypharmacy on HRQoL among US adults with arthritis. Therefore, the objective of our study was to assess the relationship between polypharmacy and HRQoL measures among US adults with arthritis.

## Methods

We conducted a longitudinal retrospective cohort study with a baseline year and follow-up year by using data from 2 longitudinal panels of the Medical Expenditure Panel Survey (MEPS). MEPS is an annual household survey of noninstitutionalized civilians (20). Interviews were conducted 5 times over the course of 2 years to minimize recall bias, providing longitudinal data (20). Computer-assisted personal interviewing technology was used to collect information on each household member. We combined data from 2 panels, panel 15 (2010–2011) and panel 16 (2011–2012) to increase sample size.

We used information provided by the households to identify medical conditions, prescription drugs used, HRQoL measures, health status, demographic and socioeconomic characteristics, employment status, and access to care (20). Chronic conditions were elicited by asking respondents to enumerate the 1) conditions in the MEPS priority list; 2) conditions leading to emergency, outpatient, inpatient, and other hospital visits; 3) conditions causing disability; and 4) conditions that bothered the respondents during a reference period (20). The study cohort consisted of adults aged over 21 years who were alive and had arthritis during the baseline year. Arthritis was identified from household or medical conditions files of MEPS. We identified adults with arthritis as those who reported having arthritis in the household file or were linked to *International Classification of Diseases, Ninth Revision* (ICD-9) codes 201, 202, 203, or 204 in the medical conditions file.

### Measures

Outcome was assessed by HRQoL by using the 12-item short form (SF-12) health survey (21). The short form measures 8 domains: general health, vitality, physical functioning, role–physical (limitations in work and daily activities because of physical problems), bodily pain, social functioning, role–emotional (limitations in work and daily activities because of emotional problems), and mental health (11,22). MEPS provides information on mental component summary (MCS) scores and physical component summary (PCS) scores. We used MCS and PCS scores to represent mental and physical health components of HRQoL. MCS and PCS scores range from 0 to 100, with higher scores representing better self-reported health and better HRQoL (11,22). The mean score of the general US population is 50 (22). The established MCS norms for the national sample of people with osteoarthritis and rheumatoid arthritis are 47.53 and 47.15, respectively, whereas the established PCS norm for people with osteoarthritis is 38.91 and for those with rheumatoid arthritis is 40.57 (22).

Polypharmacy was the key explanatory variable assessed. There is no consensus definition of polypharmacy; however, taking prescription drugs from 6 or more drug classes concurrently was a widely used definition in previous studies (23). Thus, we categorized the adults in our cohort by whether, during the baseline year, they were taking prescription drugs from 6 or more drug classes concurrently or taking prescription drugs from fewer than 6 drug classes concurrently. The MEPS prescription medications file provides information on therapeutic classes of medications through linkage with the Multum Lexicon database (http://www.multum.com/Lexicon.htm) (24). We used the unique therapeutic class codes from this database to identify the maximum number of classes of medications taken by people in the baseline year.

Other explanatory variables measured during the baseline year were sex (female, male); age (22–39 y, 40–49 y, 50–64 y, 65–74 y, ≥75 y); race/ethnicity (white, African American, Latino, or other); marital status (married, separated/divorced, widowed, or never married); poverty status based on the household’s annual income as a percentage of the federal poverty line (poor, <100%; near poor, 100% to <200%; middle income, 200% to <400%; high income, ≥400%); health insurance coverage (private, public, uninsured); prescription drug coverage (yes or no); the presence of other co-occurring physical conditions (asthma, diabetes, cancer, gastroesophageal reflux disease, heart disease, hypertension, osteoporosis, thyroid, chronic obstructive pulmonary disease), and mental conditions (depression, anxiety); baseline HRQoL measures; smoking status (current smoker or not current smoker; body mass index (BMI) (kg/m^2^) (underweight/normal [≤24.9], overweight [25.0−29.9], or obese [≥30.0]); and geographic area of residence (metropolitan or rural).

We controlled for baseline PCS in the models because a study by Fortin and colleagues suggested that lower PCS scores were associated with severity of illness for many of the chronic conditions they examined from a list of 14 anatomical domains (eg, cardiac, respiratory, renal, metabolic) (25).

### Statistical analysis

We used χ^2^ to examine the associations between polypharmacy and other explanatory variables in the bivariate analysis. The relationship between polypharmacy and HRQoL measures (MCS and PCS) was assessed by using *t* tests.

Because of the nature of the outcome, multivariable ordinary least squares (OLS) regressions were used to examine the association between polypharmacy and follow-up HRQoL measures (MCS and PCS) separately.

We constructed unadjusted and adjusted models with these as explanatory variables: number of co-existing chronic physical and mental conditions, baseline MCS and PCS scores, sex, age, race/ethnicity, marital status, poverty status, health insurance coverage, prescription drug coverage, BMI, smoking status, and geographic area of residence. In all models, the reference group was “no polypharmacy.”

All analyses accounted for the complex survey design of MEPS, and statistical testing was carried out with survey procedures in SAS, version 9.4 (SAS Institute Inc). Because of multiple comparisons, we reported all *P* values, and we considered variables with a *P* value of less than .05 to be significant.

## Results

In the combined MEPS panels, 6,132 adults had data for the baseline year and were alive during the follow-up year. We found polypharmacy among 28% of our cohort; nearly 70% of our cohort had at least 1 additional chronic physical condition. Adults with 5 or more chronic physical conditions had the highest rate of polypharmacy (89.1%). Those with 3 or 4 chronic physical conditions had a significantly higher rate of polypharmacy than those with 1 or 2 chronic physical conditions (63.2% vs 24.3%). Among adults with depression or anxiety, 45.3% took 6 or more prescription medications compared with 25.5% of adults without depression or anxiety. Adults with prescription drug coverage had significantly higher rates of polypharmacy than those without prescription drug coverage (35.9% vs 1.7%). Significant associations were found between polypharmacy and all the explanatory variables included in the study (Table 1).

**Table 1:** Characteristics of the Study Sample of Adults with Arthritis (N = 6,132), by Polypharmacy, Medical Expenditure Panel Survey (MEPS), Panels 2010–2012^a^

Characteristic	No Polypharmacy, n (Weighted %)	Polypharmacy, n (Weighted %)	*P* Value^b^
**All**	**4,436 (71.8)**	**1,696 (28.2)**	—
**Osteoarthritis**			
Yes	1,179 (66.0)	573 (34.1)	<.001
No	3,257 (74.5)	1,123 (25.5)	
**Rheumatoid arthritis**			
Yes	535** (**62.6)	299 (37.4)	<.001
No	3,901** (**73.0)	1,397 (27.0)	
**Other forms of arthritis^c^**			
Yes	2,722 (77.0)	824 (23.0)	<.001
No	1,714 (65.1)	872 (35.0)	
**Sex**			
Female	2,608 (69.3)	1,118 (30.7)	<.001
Male	1,828 (75.2)	578 (24.8)	
**Age**			
22–39	773 (92.9)	57 (7.1)	<.001
40–49	843 (84.5)	150 (15.5)	
50–64	1,583 (71.4)	643 (28.6)	
65–74	726 (60.6)	447 (39.4)	
≥75	511 (55.7)	399 (44.4)	
**Race/ethnicity**			
White	2,488 (70.7)	1,043 (29.3)	.004
African American	911 (72.8)	370 (27.2)	
Latino	749 (77.5)	196 (22.5)	
Other	288 (75.2)	87 (24.8)	
**Marital status**			
Married	2,401 (74.3)	773 (25.7)	<.001
Separated/divorced	909 (70.4)	394 (29.6)	
Widowed	433 (54.6)	365 (45.4)	
Never married	693 (79.2)	164 (20.8)	
**Poverty status^d^**			
Poor	834 (67.0)	397 (33.0)	.001
Near poor	988 (68.4)	438 (31.6)	
Middle income	1,315 (73.3)	438 (26.7)	
High income	1,299 (74.3)	423 (25.7)	
**Health insurance coverage**			
Private	2,561 (74.4)	845 (25.6)	<.001
Public	1,240 (59.4)	791 (40.6)	
Uninsured	635 (89.6)	60 (10.4)	
**Prescription drug coverage**			
Yes	3,020 (64.1)	1,672 (35.9)	<.001
No	1,416 (98.3)	24 (1.7)	
**Chronic physical conditions,^e^ no.**			
None	1,730 (96.5)	55 (3.5)	<.001
1–2	2,215 (75.7)	682 (24.3)	
3–4	466 (36.8)	764 (63.2)	
≥5	25 (10.9)	195 (89.1)	
**Chronic mental conditions,^f^ no.**			
None	3,968 (74.5)	1,334 (25.5)	<.001
≥1	468 (54.7)	362 (45.3)	
**Body mass index (kg/m^2^)**			
Underweight/normal (<24.9)	1,249 (79.7)	307 (20.3)	<.001
Overweight (25.0–29.9)	1,526 (74.5)	494 (25.5)	
Obese (≥30.0)	1,577 (64.0)	860 (36.0)	
**Smoking status**			
Current smoker	925 (75.4)	290 (24.6)	.03
All others	3,440 (71.0)	1,369 (29.0)	
**Geographic area of residence**			
Urban	3,745 (72.9)	1,342 (27.1)	.002
Rural	691 (66.7)	354 (33.3)	

Abbreviation: —, not applicable.

a Polypharmacy was defined as taking prescription drugs from 6 or more drug classes concurrently. Data were combined from 2 MEPS panels, 2010–2011 and 2011–2012, for adults aged over 21 years who reported having arthritis during the baseline year and were alive throughout the baseline and following year.

b *P* values were derived from χ^2^ tests between polypharmacy groups and explanatory variables.

c Infective arthritis and osteomyelitis (*International Classification of Diseases, Ninth Revision*, [ICD-9] code 201), other nontraumatic joint disorders (ICD-9 code 204), or reported unspecified arthritis.

d Poverty status was based on annual family income in relation to the federal poverty line. Participants were divided into 4 groups on the basis of their income’s percentage of the federal poverty line: poor (less than 100%), near poor (100% to <200%), middle income (200% to <400%), and high income (≥400%).

e Asthma, diabetes, cancer, gastroesophageal reflux disease, heart disease, hypertension, osteoporosis, thyroid disease, and chronic obstructive pulmonary disease.

f Anxiety or depression.

### HRQoL measures by polypharmacy

The mean MCS scores for the cohort during the baseline year and follow-up years were 49.35 (SE, 0.17) and 49.57 (SE, 0.18), respectively. The mean PCS scores for the cohort during the baseline and follow-up years were 42.61 (SE, 0.19) and 42.39 (SE, 0.19), respectively (Table 2). The mean MCS and PCS scores by polypharmacy during the baseline and follow-up years are listed in Table 2.

**Table 2:** Health-Related Quality of Life Measures During Baseline and Follow-up Year, by Polypharmacy^a^, Adults (N = 6,132) With Arthritis, Medical Expenditure Panel Survey, Panels 2010–2012^b^

Category	Component Summary Score, Mean (SE)	*P* Value
**Mental, baseline year**		
All	49.35 (0.17)	—
Polypharmacy	47.27 (0.30)	<.001
No polypharmacy	50.20 (0.22)	
**Mental, follow-up year**		
All	49.57 (0.18)	—
Polypharmacy	47.43 (0.32)	<.001
No polypharmacy	50.44 (0.22)	
**Physical, baseline year**		
All	42.61 (0.19)	—
Polypharmacy	35.32 (0.37)	<.001
No polypharmacy	45.58 (0.19)	
**Physical, follow-up year**		
All	42.39 (0.19)	—
Polypharmacy	35.13 (0.37)	<.001
No polypharmacy	45.34 (0.20)	

Abbreviation —, not applicable.

a Polypharmacy was defined as taking prescription drugs from 6 or more drug classes concurrently. Number in the polypharmacy group was 1,696; for the no polypharmacy group, the number was 4,436.

b Adults aged over 21 years who reported having arthritis during the baseline year and were alive throughout the baseline and following year.

Table 3 displays parameter estimates of polypharmacy from each OLS regression using MCS and PCS scores as outcomes. In the unadjusted model, adults taking prescription drugs from 6 or more drug classes concurrently had significantly lower MCS scores (β, −3.11; *P* < .001) and PCS scores (β, −10.26; *P* < .001) than those taking prescription drugs from fewer than 6.

**Table 3:** Parameter Estimates of Polypharmacy^a^ From Ordinary Least Squares Regression Outcomes Relative to Mental Component Summary (MCS) Score and Physical Component Summary (PCS) Score, Adults with Arthritis (N = 6,132)^b^, Medical Expenditure Panel Survey, Panels 2010–2012

Explanatory Variable	MCS		PCS	
	β (SE)	*P *Value	β (SE)	*P* Value
**Unadjusted model**				
Polypharmacy	−3.11 (0.41)	<.001	−10.26 (0.42)	<.001
No polypharmacy	Reference		Reference	
**Adjusted model**				
Polypharmacy	−0.27 (0.37)	.46	−1.68 (0.38)	<.001
No polypharmacy	Reference		Reference	

Abbreviation: SE, standard error.

a Polypharmacy was defined as taking prescription drugs from 6 or more drug classes concurrently.

b Adults aged over 21 years who reported having arthritis during the baseline year and were alive throughout the baseline and following year. Adjusted model included sex, age, race/ethnicity, marital status, poverty status, health insurance coverage, prescription drug coverage, number of mental and physical health conditions, body mass index (kg/m^2^), smoking status, geographic area of residence, and baseline MCS and PCS scores.

After controlling for sex, age, race/ethnicity, marital status, poverty status, health insurance coverage, prescription drug coverage, number of mental and physical health conditions, BMI, smoking status, geographic area of residence, and baseline MCS and PCS scores, adults taking prescription drugs from 6 or more drug classes concurrently had significantly lower PCS scores (β, −1.68; *P* < .001) than those taking prescription drugs from fewer than 6. However, no significant difference in MCS scores was found between adults taking prescription drugs from 6 or more drug classes concurrently and those taking prescription drugs from fewer than 6 (β, −0.27, *P* = .46).

### Sensitivity analyses

We conducted 2 sensitivity analyses that used different definitions of polypharmacy to test the robustness of the results. In the first sensitivity analysis, we defined polypharmacy as using prescription drugs from 4 or more drug classes concurrently. In the second sensitivity analysis, we defined polypharmacy as using drugs from 5 or more drug classes. In both analyses, the negative associations between polypharmacy and PCS scores remained significant and consistent with the primary analyses.

With polypharmacy defined as taking prescription drugs from 4 or more drug classes concurrently, the unadjusted analyses indicated negative associations between polypharmacy and MCS (β, −2.72; *P* < .001) and PCS (β, −9.50; *P* < .001) scores. In the adjusted model, adults taking prescription drugs from 4 or more drug classes concurrently had significantly lower PCS scores than those taking prescription drugs from fewer drug classes (β, −1.71; *P* < .001). However, we found no difference in MCS scores between adults taking prescription drugs from 4 or more drug classes concurrently and those taking prescription drugs from fewer drug classes (β, −0.31; *P* = .37).

With polypharmacy defined as taking prescription drugs from 5 or more drug classes concurrently, unadjusted models indicated negative associations between polypharmacy and MCS (β, −3.00; *P* < .001) and PCS scores (β, −9.85; *P* < .001). In the adjusted model, adults taking prescription drugs from 5 or more drug classes concurrently had significantly lower PCS scores than those taking prescription drugs from fewer drug classes (β, −1.87; *P* < .001). We found no difference in MCS scores (β, −0.31; *P* = .39).

## Discussion

Our study examined the relationship between polypharmacy and HRQoL among adults with arthritis. Among our cohort of US adults with arthritis, 28% were found to be taking prescription drugs from 6 or more drug classes concurrently. The average PCS scores for these adults were below the established PCS norms for people with osteoarthritis and rheumatoid arthritis, suggesting that adults in our cohort had poorer health than the general US population with osteoarthritis or rheumatoid arthritis. We also found that more adults taking prescription drugs from 6 or more drug classes concurrently had 3 or more chronic physical conditions compared with those taking prescription drugs from fewer drug classes. After controlling for other explanatory variables, adults taking prescription drugs from 6 or more drug classes concurrently had significantly lower PCS scores than those taking prescription drugs from fewer drug classes.

Our results suggest that polypharmacy is negatively associated with PCS scores among adults with arthritis. Our findings are consistent with those of previous studies showing that polypharmacy is associated with low PCS scores among older American Indians (18), rural elderly (19), and male veterans (17).

Nearly 70% of the adults in our cohort had 1 or more chronic physical conditions in addition to arthritis. The management of arthritis often requires multiple prescription medications, including analgesics, anti-inflammatory drugs, corticosteroids, disease-modifying antirheumatic drugs, or biological therapies (12,13); co-existing conditions may need to be treated with drugs, placing adults with arthritis at high risk for polypharmacy. Polypharmacy is associated with drug–drug and drug–disease interactions that may lead to development of new diseases or worsen control of existing diseases. Therefore, health care providers need to weigh the harms and benefits before prescribing multiple drugs for the management of arthritis and other co-occurring conditions. In addition, further investigation of the impact of the different drug classes on the PCS measure is needed. Identifying the drug classes that are associated with lower PCS scores was beyond the scope of this study.

In the adjusted analyses, we found no differences in the MCS scores between adults taking prescription drugs from 6 or more drug classes concurrently and those taking prescription drugs from fewer drug classes. This is consistent with the results of a previous study showing that polypharmacy is not associated with MCS scores among older American Indians (18). Nevertheless, the average MCS scores for adults in our study cohort were above the established MCS norms for people with osteoarthritis and rheumatoid arthritis (22).

To the best of our knowledge, this is the first study that assesses the relationship between polypharmacy and HRQoL measures among adults with arthritis by using a nationally representative data set and well-established and validated measures of HRQoL. We controlled for a comprehensive list of factors that may affect HRQoL. Availability of information on prescription drug classes enabled us to measure polypharmacy. However, this study had limitations. First, information on all the variables was based on self-reported data, which increases the risk for recall bias. Second, we could not control for the severity of arthritis in the baseline year. Third, MEPS does not provide information on nonprescription drugs, such as analgesics and anti-inflammatories. Finally, we did not explicitly control for arthritis-related treatment that may affect HRQoL.

Polypharmacy is significantly associated with lower PCS scores among adults with arthritis. Further research is required to identify the drug classes associated with low PCS scores. Because polypharmacy can lead to drug–drug and drug–disease interactions, health care providers should assess risk and adopt a cautious approach in prescribing multiple drugs to manage chronic conditions and should choose therapies that improve HRQoL among adults with arthritis.
